# Implementation and evaluation of nonclinical interventions for appropriate use of cesarean section in low- and middle-income countries: protocol for a multisite hybrid effectiveness-implementation type III trial

**DOI:** 10.1186/s13012-020-01029-4

**Published:** 2020-09-04

**Authors:** Alexandre Dumont, Ana Pilar Betrán, Charles Kaboré, Myriam de Loenzien, Pisake Lumbiganon, Meghan A. Bohren, Quoc Nhu Hung Mac, Newton Opiyo, Guillermo Carroli, Kristi Sidney Annerstedt, Valéry Ridde, Ramón Escuriet, Michael Robson, Claudia Hanson, The QUALI-DEC research group, Claudia Hansen, Helle Molsted-Alvesson, Kristi Sidney Annerstedt, Michael Robson, Ana Pilar Betràn, Newton Opiyo, Meghan Bohren, Guillermo Carroli, Liana Campodonico, Celina Gialdini, Berenise Carroli, Gabriela Garcia Camacho, Daniel Giordano, Hugo Gamerro, Mariana Romero, Pisake Lumbiganon, Dittakarn Boriboonhirunsarn, Nampet Jampathong, Kiattisak Kongwattanakul, Ameporn Ratinthorn, Olarik Musigavong, Ramon Escuriet, Olga Canet, Charles Kabore, Yaya Bocoum Fadima, Simon Tiendrebeogo, Zerbo Roger, Mac Quoc Nhu Hung, Thao Truong, Tran Minh Thien Ngo, Bui Duc Toan, Huynh Nguyen Khanh Trang, Hoang Thi Diem Tuyet, Alexandre Dumont, Laurence Lombard, Myriam de Loenzien, Marion Ravit, Delia Visan, Angela Hermann, Valéry Ridde

**Affiliations:** 1 CEPED, Institute for Research on Sustainable Development, IRD-Université de Paris, ERL INSERM SAGESUD, Paris, France; 2 UNDP/UNFPA/UNICEF/World Bank Special Program of Research, Development and Research Training in Human Reproduction (HRP), Department of Sexual and Reproductive Health and Research, World Health Organization, Geneva, Switzerland; 3 Institut de Recherche en Sciences de la Santé, Ouagadougou, Burkina Faso; 4 Department of Obstetrics and Gynaecology, Faculty of Medicine, Khon Kaen University, Khon Kaen, Thailand; 5 Gender and Women’s Health Unit, Centre for Health Equity, School of Population and Global Health, University of Melbourne, Melbourne, Australia; 6 Pham Ngoc Thach University, Ho Chi Minh City, Vietnam; 7 Centro Rosarino de Estudios Perinatales, Rosario, Argentina; 8 Department of Public Health, Karolinska Institutet, Stockholm, Sweden; 9 Fundacio Blanquerna, Barcelona, Spain; 10 University College Dublin, National University of Ireland, Dublin, Ireland; 11 Department of Global Public Health, Karolinska Institutet, Stockholm, Sweden; 12 London School of Hygiene and Tropical Medicine, London, UK

**Keywords:** Unnecessary cesarean section, Quality of care, Shared decision-making, Nonclinical intervention, Healthcare organization, Low- and middle-income countries

## Abstract

**Background:**

While cesarean sections (CSs) are a life-saving intervention, an increasing number are performed without medical reasons in low- and middle-income countries (LMICs). Unnecessary CS diverts scarce resources and thereby reduces access to healthcare for women in need. Argentina, Burkina Faso, Thailand, and Vietnam are committed to reducing unnecessary CS, but many individual and organizational factors in healthcare facilities obstruct this aim. Nonclinical interventions can overcome these barriers by helping providers improve their practices and supporting women’s decision-making regarding childbirth. Existing evidence has shown only a modest effect of single interventions on reducing CS rates, arguably because of the failure to design multifaceted interventions effectively tailored to the context. The aim of this study is to design, adapt, and test a multifaceted intervention for the appropriate use of CS in Argentina, Burkina Faso, Thailand, and Vietnam.

**Methods:**

We designed an intervention (QUALIty DECision-making—QUALI-DEC) with four components: (1) opinion leaders at heathcare facilities to improve adherence to best practices among clinicians, (2) CS audits and feedback to help providers identify potentially avoidable CS, (3) a decision analysis tool to help women make an informed decision on the mode of birth, and (4) companionship to support women during labor. QUALI-DEC will be implemented and evaluated in 32 hospitals (8 sites per country) using a pragmatic hybrid effectiveness-implementation design to test our implementation strategy, and information regarding its impact on relevant maternal and perinatal outcomes will be gathered. The implementation strategy will involve the participation of women, healthcare professionals, and organizations and account for the local environment, needs, resources, and social factors in each country.

**Discussion:**

There is urgent need for interventions and implementation strategies to optimize the use of CS while improving health outcomes and satisfaction in LMICs. This can only be achieved by engaging all stakeholders involved in the decision-making process surrounding birth and addressing their needs and concerns. The study will generate robust evidence about the effectiveness and the impact of this multifaceted intervention. It will also assess the acceptability and scalability of the intervention and the capacity for empowerment among women and providers alike.

**Trial registration:**

ISRCTN67214403

Contributions to the literature

- Overuse of cesarean section diverts essential resources and has a negative impact on maternal or child health.
- Despite the range of evidence-based interventions, the research reported to date has shown only modest effectiveness in reducing cesarean section rates.
- The QUALI-DEC project acknowledges the multifactorial and complex nature of overuse of cesarean section.
- It combines different interventions across stakeholders and focuses on how to best and most effectively implement them, accounting for the local context.
- The research will add if combination of interventions at women’s and health systems level can contribute to optimizing the use of cesarean section and why and how the intervention works.

## Background

Despite the short- and long-term risks associated with cesarean section (CS) [1], the proportion of births by CS continues to increase [2]. This trend is not confined to high-income countries, but it widely affects low- and middle-income countries (LMICs), where the overuse and underuse of CS coexist, widening health inequalities and diverting scarce resources [3]. When clinically indicated, a CS can effectively prevent maternal and perinatal mortality and morbidity; however, there is no evidence of the benefits of a CS for women and infants who do not need the procedure, and as with any surgery, there are risks associated that are higher in LMIC settings [4–6]. Women with a single fetus in cephalic presentation who have reached at least 37 weeks’ gestation and with no previous CS—a group considered low risk—are major contributors to the growing prevalence of CS [7]. This pattern has also been described in LMICs. Unnecessary CS may be particularly prevalent among low-risk women since these women account for approximately half of all CS.

The present evidence on care for women during childbirth has been summarized in the World Health Organization (WHO) recommendations on intrapartum care for a positive childbirth experience [8] and may enhance the appropriate use of CS if used systematically by healthcare professionals. However, the overuse of CS can no longer be seen only as the result of suboptimal clinical practices during childbirth. Nonclinical factors, such as social, cultural, and organizational influences, have emerged as potential drivers and need to be considered to effectively optimize the use of CS [9]. Nonclinical interventions that address these factors are defined as those applied independently of a clinical encounter between a healthcare provider and a woman in the context of patient medical care and have been shown to safely reduce CS rates, predominantly in high-income settings [10]. They may target providers who are involved in CS decision-making (physicians, nurses, and midwives), women and families, or healthcare organizations or facilities.

**Fig. 1:**
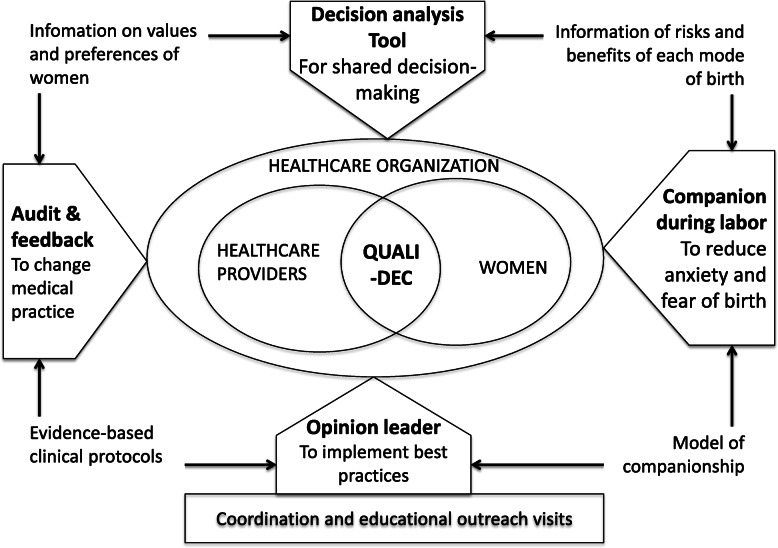
Quality decision-making (QUALI-DEC) by women and healthcare providers for appropriate use of cesarean section

The effectiveness of nonclinical interventions to reduce unnecessary CS has also been summarized by the WHO [10, 11]. Based on randomized controlled trials with moderate- to high-certainty evidence (Table 1), we designed a multifaceted intervention called QUALI-DEC to improve decision-making regarding CS (appropriate use of cesarean section through QUALIty DECision-making by women and providers), four components constitute QUALI-DEC: (1) Opinion leaders (OLs) at healthcare facilities to implement best practices, (2) CS audits and feedback to help providers identify areas for improvements in medical practices; (3) a decision analysis tool (DAT) to help women make an informed decision on the mode of birth, and (4) companionship to support women during labor. The theoretical framework shown in Fig. 1 and Table 2 describe how these four mutually reinforcing components may reduce unnecessary CS by improving the decision-making of women and providers regarding the mode of birth.

**Table 1:** Published randomized controlled trials with moderate- to high-certainty evidence

Study	Study design	Type of intervention	Overall CS rate in %				Relative effect (95% CI)
			Intervention		Control		
			Baseline	Post	Baseline	Post	
Lomas [12]	Cluster RCT	Opinion leader education		53.7*		66.8*	Not reported
		Audit and feedback		69.7*		66.8*	
Althabe [13]	Cluster RCT	Mandatory second opinion	26.3	24.7	24.6	24.9	ARR −1.9 (−3.8 to −0.1)
Chaillet [14]	Cluster RCT	Audit and feedback	22.5	21.8	23.2	23.5	ARR −1.8 (−3.8 to −0.2)
	RCT		8.5**	7.6**	8.5**	9.0**	ARR −1.7 (−3.0 to −0.3)
Mansoumi [15]	RCT	Antenatal education program for physiologic childbirth		45.0		43.7	RR 1.03 (0.72 to 1.49)
Bergstrom [16]	RCT	Antenatal education on natural childbirth preparation with training in breathing and relaxation techniques		59.9*		63.0*	RR 0.95 (0.58 to 1.56)
Fraser [17]	RCT	Individualized prenatal education and support program versus written information in pamphlet		21.3		23.7	RR 0.90 (0.74 to 1.11)
Montgomery [18]	RCT	Computer-based decision aids (information program, decision analysis)		48.6*		49.6*	RR 0.98 (0.82 to 1.18)
Shorten [19]	RCT	Decision aid booklet during antenatal care		49.4*		52.2*	Not reported
Bohren [11]	Meta-analysis	Companionship during labor		12.3		15.0	RR 0.75 (0.64 to 0.88)

*RCT* randomized controlled trial with intervention at the woman’s level; *cluster-RCT* randomized controlled trial with intervention at the hospital or healthcare provider level

For RCTs, risk ratio (RR) = (mean rate intervention/mean rate control) with 95% confidence intervals

For the meta-analysis of RCTs, the relative effect is the summary risk ratio with 95% confidence intervals

For cluster-RCTs, absolute risk reduction (ARR) = (rate change in the intervention group)—(rate change in the control group) with 95% confidence intervals

*The selected outcome is the elective repeat cesarean section rate among high-risk women (women with previous CS)

**The selected outcome is the overall CS rate among low-risk women (single pregnancy with cephalic presentation without any complication)

**Table 2:** Definition, theory, and assumptions of each component of the QUALI-DEC intervention

Component	Definition	Theoretical stance	Assumption
Opinion leaders [20]	Healthcare leaders are identified by their colleagues or local authorities in participating healthcare facilities as being respected clinicians and effective communicators.	Power/interaction model of interpersonal influence [21]	Adherence to guidelines and clinical audit are reinforced through the interaction and influence of reputable culture change agents.
Audit and feedback [22]	Indications of CS and CS practice among low-risk women are audited by a local committee, with timely feedback to all healthcare professionals.	Constructivist learning [23]	The way knowledge is absorbed, processed, and retained results from cognitive, emotional, and environmental influences, and change occurs through the active involvement of professionals in analyzing their practices.
Decision analysis tool (DAT) [24]	A meaningful dialog between providers and women on preferences, options, concerns, risks and benefits of planned CS vs. planned vaginal delivery leads to an informed and more satisfactory decision for both parties.	Decision theory [25]	A decision aid benefits women and healthcare workers by facilitating a process of informed decision-making, in the context of improved knowledge and overt consideration of women’s individual fears, values, and needs surrounding birth.
Companionship during labor [11]	Through the process of implementation, professionals decide on the modification of existing systems, structures, or tasks to offer women and their relatives the possibility of having a companion of choice during labor and childbirth.	Convoy model of social relations [25, 26]	Overuse of CS can be prevented by improving the design of health systems and processes to better respond and adapt to the needs of women and their relatives regarding social support during labor and childbirth.

In this study, we will design, adapt, and evaluate a strategy to implement the four components of the QUALI-DEC intervention. Our primary hypothesis is that the implementation of quality decision-making supported by a local OL, continuous CS audit and feedback, use of the DAT during antenatal care, and companionship during labor could reduce CS rates among low-risk women. The specific objectives of the study are as follows:

1. To evaluate the QUALI-DEC strategy at the health professional and health system levels in terms of participation, acceptability, implementation, scalability, and empowerment of providers through the audit approach, costs at the organization level, and scalability.
2. To evaluate the QUALI-DEC strategy at the women’s level in terms of participation in activities targeting them, acceptability, scalability, and the empowerment of women in decision-making regarding the planned mode of birth and satisfaction with care.
3. To assess the effect of the multifaceted intervention on CS rates and maternal and perinatal outcomes.
4. To conduct extended cost-effectiveness analyses of implementing QUALI-DEC interventions from women’s perspective and the health system perspective, using both health and nonhealth outcomes.

## Methods: Description

### Study design

We will use a pragmatic hybrid effectiveness-implementation type III [12] design to test our implementation strategy while observing and gathering information on the QUALI-DEC intervention’s impact on relevant outcomes. Using a quasi-experimental design (interrupted time series and before-after study), we will assess effectiveness and safety outcomes [13, 14]. A process evaluation will be carried out using mixed qualitative and quantitative approaches [15]. We used the Standards for Reporting Implementation Studies (STaRI) checklist to report our research protocol [16].

### Context

The multifaceted intervention will be implemented in facilities in Argentina, Burkina Faso, Thailand, and Vietnam. These four countries illustrate various degrees of rates in LMICs (Table 3) and present specific challenges for QUALI-DEC implementation. Within these four countries, Argentina has the highest level of CS at the national level and, more generally, of the biomedicalization of childbirth. Thailand has very low fertility, which may add pressure in favor of CS. A favorable socioeconomic context may also facilitate the preference for CS. Vietnam is interesting for its demographic impact (size of the population) and its performance in health indicators given its level of national income. However, the national CS rate has been continuously increasing over the past few decades, exceeding any reasonable level for medical needs and large inequalities in the use of CS. Burkina Faso has a low CS rate at the national level that may hide inequalities [17] and that suggests a great potential for further increase and consequently represents an opportunity to prevent the phenomenon before it aggravates.

**Table 3:** Main health indicators at country level

Indicator, 2017-2019*	Argentina	Burkina Faso	Thailand	Vietnam
Population (millions)	44.9	20.3	66.4	95.7
Total fertility rate	2.3	5.3	1.5	2.0
Maternal mortality ratio	39	320	37	43
Neonatal mortality rate	6.4	24.7	5.0	10.6
Institutional delivery rate	100%	80%	99%	94%
Cesarean section rate	36%	3%	33%	27%
Risk of impoverishing expenditure for surgical care	3.9%	75.9%	6.3%	27.4%
GDP per capita (PPP international $) 2018	20,611	1985	19,051	7478
Income group of the country	Upper-middle income	Lower income	Upper-middle income	Middle income

*Latest estimation according to the following source of information: (1) WHO Statistical Information System : https://www.who.int/whosis/indicators/en/; (2) World Bank national accounts data: https://data.worldbank.org/indicator/NY.GDP.PCAP.PP. CD

Maternal mortality ratio: number of maternal deaths per 100,000 live births

Neonatal mortality rate: number of newborn deaths per 1000 live births

Impoverishing expenditure is defined as direct out-of-pocket payments for surgical and anesthesia care which drive people below a poverty threshold (using a threshold of $1.25 PPP/day).

Risk of impoverishing is the proportion of population at risk of impoverishing expenditure when surgical care is required

### Targeted sites and participants

The study will be conducted from January 2020 to December 2024, in 32 healthcare facilities (8 per country) with high CS rates. Facilities were selected purposely with country investigators to reflect the range of contexts, such as secondary and tertiary levels of care, public and private hospitals, and teaching and nonacademic facilities (Table 4). The intervention directly targets healthcare providers involved in obstetric care and all women who give birth in the participating hospitals during the study period. We have defined providers as obstetricians and nurses/midwives working in the maternity ward in the study facilities. Women will be eligible if they give birth to a newborn (birthweight ≥ 500 g in Argentina and Vietnam or ≥ 1000 g in Burkina Faso and Thailand), alive or dead, and with or without malformations. The intervention does not target patients admitted for abortion or miscarriage or those who delivered at home or in another facility that is not a participating hospital.

**Table 4:** Characteristics of participating hospitals by country

Characteristic	Argentina	Burkina Faso	Thailand	Vietnam
Type of hospital				
Public without private ward	8	8	0	2
Public with private wards	0	0	8	4
Private	0	0	0	2
Level of reference				
Tertiary	4	2	6	2
Secondary	4	4	2	4
Primary	0	2	0	2
Teaching hospital				
Yes	8	3	8	4
No	0	5	0	4
Type of medical records				
Electronic	8	0	4	1
Paper-based	0	8	4	7
Range of annual births	1200-5600	2500-6000	2500-7500	2800-42,000
Range of CS rates	23-38%	21-48%	36-56%	23-54%

### Intervention

A multifaceted intervention was developed based on existing evidence (Table 1) and WHO recommendations on nonclinical interventions to reduce unnecessary CS [18]. Baseline formative research [19] informed by the ecological framework [9] will be conducted to improve our understanding of the different levels of factors affecting CS rates and to adapt the multifaceted intervention to each country. The four components of QUALI-DEC will be implemented simultaneously in each participating hospital during the 2-year implementation period (Fig. 1):

Component 1—opinion leader (OL)—one OL in each facility has been identified by peers and local authorities. OLs are gynecologists-obstetricians with proven communication skills and a reputable influence on their colleagues. The OLs will take part in a 5-day training session at the beginning of the implementation period. This training will include 1 day training for each of the following topics: (1) mobilizing OLs on the power/interaction model of interpersonal influence; (2) selecting evidence-based clinical protocols for CS decision-making; (3) audit and feedback including external review of medical records and use of Robson classification as a feedback tool; (4) use of decision-analysis tool; and (5) implementing continuous companionship during labor. After the initial training, OLs will create local committees, launch the audit and feedback, and encourage the use of the DAT and companionship during labor in their own hospitals. OLs will undergo a refresher 3-day training session during the 2-year intervention period. The aim of this session is to refresh OLs’ knowledge, update them on the use of evidence-based clinical guidelines and process of the intervention, discuss their roles, share their experiences, and confirm their capacity to provide leadership in their clinical settings.

**Fig. 2:**
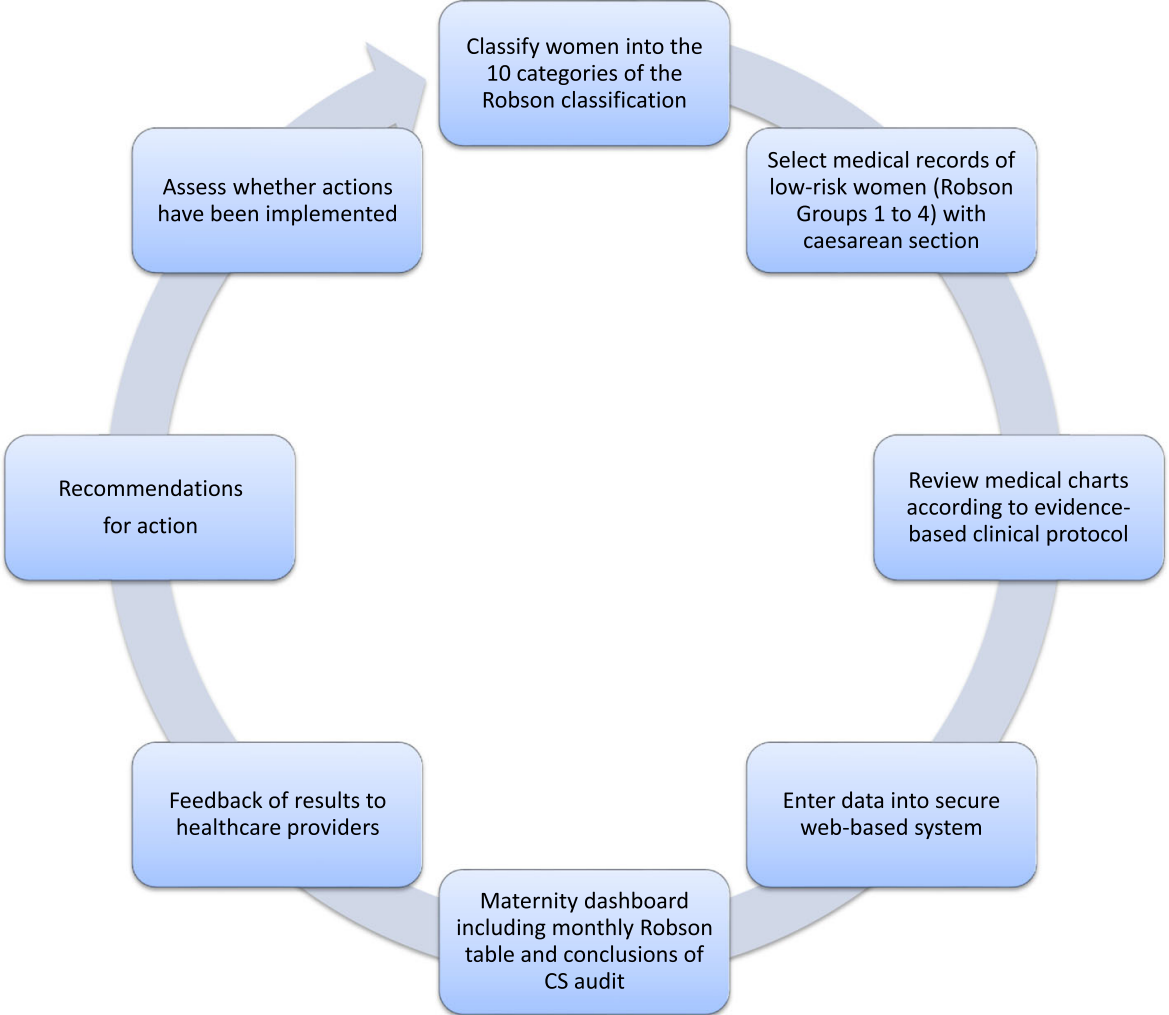
Audit cycle to change medical practice

Component 2—audit and feedback (A&F)—audit cycles will be implemented monthly by the local committees following the different steps presented in Fig. 2. Local data collectors will prospectively identifies groups of women who are admitted for childbirth using the Robson classification system ^20^. Then, medical records of low-risk women (Robson group 1 to 4) will be selected to audit the indications for cesarean sections. The local committee will provide a conclusive analysis that will be presented to the rest of the medical staff (feedback). It will allow for comparison and analysis of cesarean section rates within and across the different groups of women, as well as comparisons to other facilities. Additionally, it will help to ensure that cesarean sections are performed for clinically valid reasons, and identify priority areas for coaching, training, and support for healthcare providers.

Component 3—decision analysis tool (DAT)—the DAT is adapted to each country and developed to be used during antenatal care (after 28 weeks of gestation) by women with a singleton pregnancy, without a previous CS and eligible for a trial of labor. It includes two sections: (i) an information section, providing a description and an explanation of the risks and benefits of each mode of birth (planned vaginal birth vs. planned CS); and (ii) an exercise section, allowing women to clarify and summarize their values and preferences with their clinician and indicate what aspects of the mode of birth are important to them. The DAT will be available as a paper booklet and an interactive web/smartphone application. The DAT is designed to supplement regular counseling and discussions with healthcare providers. They will provide detailed, specific, and personal options and outcomes in order to prepare women to make the decision about the mode of delivery.

Component 4—companionship during labor—the companion can be any person chosen by the woman to provide her with continuous support during labor and childbirth. This may be someone from the woman’s family or social network, such as her spouse/partner, a female friend or relative, a community member (such as a female community leader, health worker, or traditional birth attendant) or a doula (i.e., a woman who has been trained in labor support but is not part of the healthcare facility’s professional staff). The QUALI-DEC strategy will support the use of any type of culturally appropriate companion who the woman has selected. This component will be implemented using a tailored labor companionship model that will include information on (1) eligibility criteria for women and companions, (2) identification of healthcare providers who will invite the chosen and eligible labor companion from the waiting area into the labor room, (3) identification of healthcare providers who will deliver the messages to the laboring woman and her companion, (4) how many people are allowed and when they are allowed to act as companions, (5) how physical space of the labor ward may need design modifications to accommodate a companion, and (vi) educational tools for companions on how to support women during labor and birth.

### Implementation strategy

The implementation strategy is aligned within the usual model of care in participating healthcare facilities. The main implementers are the local OLs and healthcare providers who are involved in the program and are supported by the country-level study coordinator. Formative research in the baseline period will assess the main drivers and barriers, and a meeting will be held among all stakeholders to discuss implementation issues. Parliamentarians and representatives of women’s associations will be involved in this meeting to consider women’s views. Then, the intervention will be introduced in each country with the 5-day training workshop addressed to OLs. OLs will receive financial incentives during the intervention period to compensate for the loss of revenues related to the decrease in their clinical activities. OL supported by local committee will encourage antenatal care providers to deliver the DAT booklet to eligible pregnant women. This will require a series of on-site meetings in all relevant facilities to inform and motivate providers and to obtain their formal commitment. In addition, a DAT application will be developed for smartphones and made available in the settings in which it is considered culturally appropriate and most acceptable and convenient for women. Posters will be displayed on the wall of the waiting room of antenatal care centers with the QR code to access the web/smartphone application. Other information, educational and communication (IEC) materials, such as flipcharts or posters, will be developed to facilitate the briefing of healthcare providers, companions, and laboring women. These IEC materials will include reminders about the importance of labor companionship, the role of companions, and the regulations of the labor wards. The country-level coordinator will conduct quarterly visits to each participating hospital during the 2-year implementation period to identify further barriers for the implementation process and possible strategies to overcome those barriers, verify data quality and document and report on the study’s progress.

## Methods: Evaluation

### Outcomes

The primary endpoint measure is the monthly CS rate in participating hospitals among women with a singleton pregnancy, with a fetus in cephalic presentation and at least 37 weeks of gestation, and with no previous CS (groups 1-4 of the Robson classification). We will use the Robson classification to monitor CS rates at the hospital level [7, 27, 28]. This system classifies women into prospective mutually exclusive and totally inclusive groups of women based on a few obstetric variables which are easily obtained and most women themselves would know. Trained data collectors will gather information about each eligible woman using existing routine health information systems (paper-based or electronic records). We will consider the monthly rate of CS before the onset of intervention (12-month period), during the implementation phase (24-month period), and after the implementation phase (24-month period) to assess the effects of the intervention.

As secondary endpoints, the following outcome measures will be assessed: assisted vaginal delivery; time of CS (before or during labor); third- or fourth-degree perineal laceration; antibiotics and uterotonics use; transfusion; admission of the mother or the newborn to intensive care unit; uterine rupture, hysterectomy, maternal or neonatal death; time of breastfeeding initiation; woman’s satisfaction with care and her birth experience; payment for medical care; indirect costs of care for childbirth (e.g., cost of transportation to hospital); and loss of earnings. A cross-sectional survey among a representative sample of postpartum women will be established at two time points: at baseline and at the end of the intervention period. All births occurring in the participating hospitals during 2 weeks in Argentina and Burkina Faso and 1 week in Thailand and Vietnam will be covered in each survey. The data collection includes a face-to-face interview with women after childbirth and before they leave the maternity ward (facility-exit interview) and the collection of information from the women’s medical records, including socioeconomic characteristics of the mother, reproductive history, antenatal and intrapartum care, time and indication of CS, if any, satisfaction with birth experience, breastfeeding practices, out-of-pocket costs, maternal and neonatal outcomes.

### Process evaluation

**Fig. 3:**
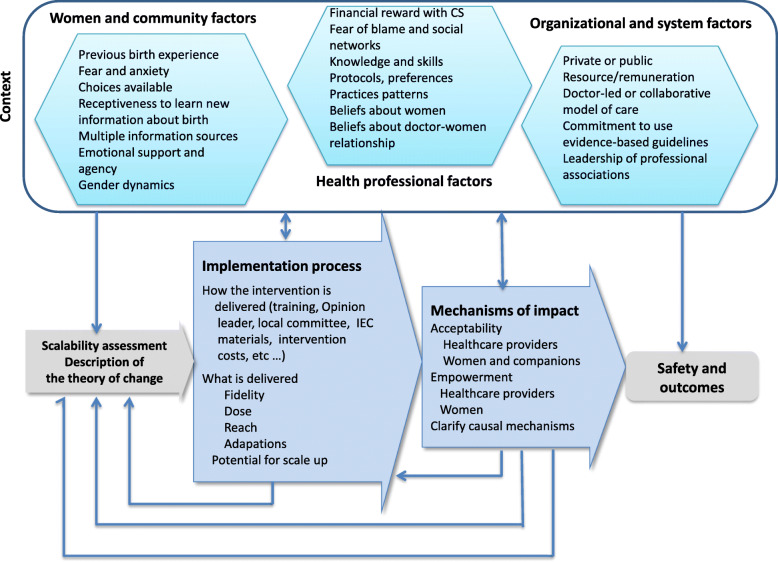
Key functions of the process evaluation and the relations among them (adapted from Moore 2015) [15]

**Fig. 4:**
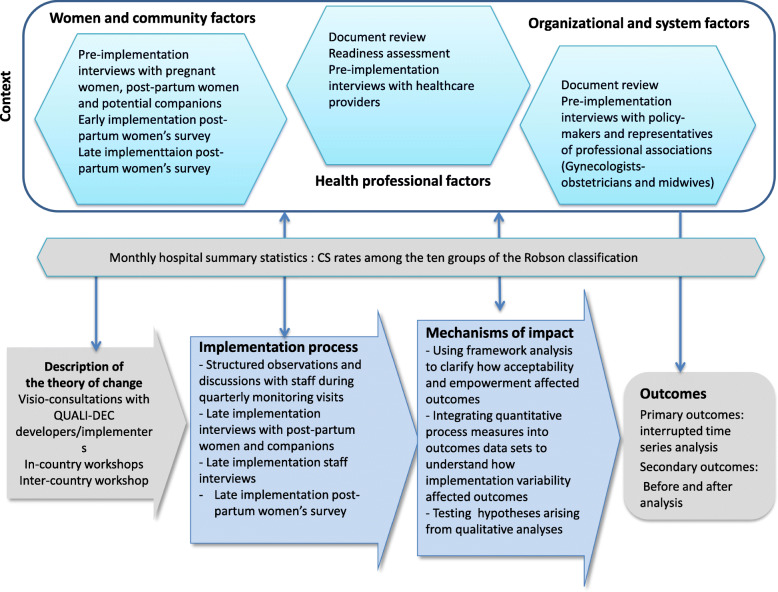
Data collection and analysis methods for process evaluation

We will use the UK Medical Research Council (MRC) process evaluation framework [15] to describe how the intervention works (or does not work) along the pathway of implementation, including the internal dynamics of the four components of the QUALI-DEC strategy. The process evaluation also explores the roles, perceptions, and coping strategies of actors, adaptation of the interventions based on the local context, and any unintended effects, with a view to understand the mediating effect of the context [29]. Figure 3 presents the key functions of the QUALI-DEC process evaluation and the relations among them, while Fig. 4 shows the data collection and analysis methods.

To align the intervention and the implementation strategy to the local context, we conduct qualitative research, document review at the country level, and a readiness assessment of each participating facility. The qualitative research will include semi-structured interviews with women, potential companions, and healthcare providers to obtain a comprehensive understanding of the health system and societal context in each country [19]. Additional interviews with policy-makers and representatives of women’s and professional associations (gynecologists-obstetricians and nurses/midwives) will allow us to complete the stakeholder mapping and analysis. Following the context assessment and in consultation with QUALI-DEC developers and implementers, we will define assumptions on what may need to happen (mechanisms of change), and we will hypothesize about how change will happen at the individual level (healthcare providers, women, and companions), at the organizational level (healthcare facility), and through the interaction of participants. Discussions and meetings will be held by video conference with country researchers and during face-to-face meetings with high-level stakeholders and local OLs in each country. To further assess the potential scalability of the intervention within the countries, we will include a participative scalability assessment in the in-country meetings to summarize early opportunities and challenges for scale up [30].

We will construct a theory of change to guide the process evaluation. We will define indicators of fidelity (whether the intervention was delivered as intended), dose (the quantity of intervention implemented), and reach of intervention (whether women and providers came into contact with each relevant component of QUALI-DEC) [15]. These indicators will be measured at the individual and organizational levels in all facilities using quarterly monitoring visits at each hospital and data from the postpartum cross-sectional survey (proportion of women who used the DAT during antenatal care or chose a companion during labor).

We will conduct in-depth case studies in a subset of four hospitals per country to investigate the details of what worked, why, and why not. Study sites will be purposively selected based on indicators of fidelity, dose, and reach to reflect the diversity of implementation across participating hospitals. Structured observations of each site and on-site meetings will be held with the members of the local committee, maternity ward staff, and facility administrator. In-depth interviews with providers and women will provide more detailed information on the perceptions and views of both stakeholders. The study instrument for IDIs with healthcare providers will be a semistructured interview guide covering the following topics: communication; interprofessional interaction; acceptability of the CS audit and feedback, DAT and labor companionship; and decision-making, including aspects of position/seniority, gender, weighing of alternatives and their implications, and information-sharing. The study instrument for IDIs with women and their companions will be a semistructured interview guide covering the following topics: process of and factors affecting the decision-making to use DAT and labor companionship; perceptions of the DAT and labor companionship related to knowledge, experiences, and support in choosing the mode of childbirth; perceptions and experiences of the relationship between themselves and providers; perception and experiences of how use of the DAT and/or labor companionship influenced trust, self-esteem, empowerment, and the relationship with providers. All interviews will take place in a private setting and will be audio recorded.

### Economic evaluation

The cost-effectiveness of the QUALI-DEC intervention and the financial risk protection provided are important factors for decision-makers considering implementing new strategies to reduce unnecessary CS. We will use an extended cost-effectiveness analysis (ECEA) approach to evaluate the cost-effectiveness of the QUALI-DEC intervention [31]. The impact of QUALI-DEC will be estimated in three domains across women in distinct wealth strata: (i) health gains (e.g., reduced CS rates), (ii) women’s out-of-pocket (OOP) expenditures averted by reducing unnecessary CS, and (iii) total net cost of the intervention to the implementer (see Additional files 1, 2 and 3).

### Sample size

Limited guidance is available on sample size calculation for time series analyses, and many of the recommendations focus on the need for sufficient time points pre- and postintervention to precisely ascertain trends and levels [32, 33]. Our analysis on the primary outcome (monthly CS rates among low-risk women) will include 12 time points preintervention, 24 time points during the intervention phase, and 24 time points during the follow-up phase.

The approach for calculating the required number of participants for the postpartum cross-sectional survey is used for a “before-after” noncontrolled study design. We estimated that a sample of 470 women at baseline and 470 women at the end of the intervention period will ensure 90% statistical power to detect an effect size of 0.3 standard deviations or greater in satisfaction scores with a two-sided 5% significance level [34]. The calculation accounted for the clustered nature of the data by hospitals with a design effect of 2. Allowing for a 20% nonresponse rate, we aim to recruit 564 women in each phase. The proposed sample size (i.e., 564) can be achieved with 2 weeks of data collection in Argentina and Burkina Faso and with 1 week of data collection in Thailand and Vietnam. This sample size will be for each country and will allow to draw conclusions independently for each country and produce individual country interpretations. We estimated that 3980 births will occur during this data collection period in all participating hospitals of the four countries. This overall sample size for the cross-sectional survey will ensure accurate measurements of other secondary outcomes (maternal and perinatal morbidity, time of breastfeeding initiation). Estimations of outcomes at each time point will fluctuate within a 95% confidence interval with the following bounds: 10% rate ± 1% (example: postpartum hemorrhage); 20% rate ± 1.3% (example: second-degree perineal trauma); 40% rate ± 1.5%; (examples: overall CS rate, breastfeeding within 1 h of birth).

### Analysis

#### Quantitative analysis

We will use different methods to evaluate the effectiveness of the QUALI-DEC intervention [13, 14]. For the primary outcome, interrupted time series analysis (ITSA) based on segmented regression will estimate the mean changes in the level (immediate change) and trend (sustained change) of monthly CS rates across all participating hospitals in relation to their baseline level and pre-existing trend. We will follow the quality criteria proposed by Ramsay et al. for our ITSA to ensure that our study is adequately reported [35]. For secondary outcomes, we will use a before and after cross-sectional design that will include medical records and women’s interviews. We will compare the outcomes between the two periods of the cross-sectional survey, adjusting for hospital and woman characteristics, to evaluate changes in satisfaction with the birth experience, breastfeeding and medical practices, and maternal/perinatal morbidity. For implementation outcomes, checklist ratings during monitoring visits will be used to compute average scores of the fidelity, dose, and reach of the intervention. Parametric tests will be used to assess changes over time for each facility and differences between study sites.

#### Qualitative analysis

Qualitative data from observations and IDIs will be analyzed and interpreted using a thematic analysis approach [36]. Interview transcripts will be analyzed in the local language at the country level. Final themes and key quotations will be translated into English for sharing with the researchers of the QUALI-DEC consortium. Framework analysis will be used to provide an in-depth understanding of acceptability [37, 38] by providers and women/companions and the empowerment [39, 40] of both stakeholders to act on CS decision-making. We will define acceptability as the perception of providers and women that the QUALI-DEC intervention is agreeable, entails an acceptable burden, is ethical and economically feasible, and leads to positive outcomes. We aim to understand the extent to which each component of the intervention and its process are both socially and technically accepted in each context. Empowerment can be understood not only as a process but also as an outcome to assess whether the intervention has helped providers and women act on CS decision-making. The analysis will focus on the individual empowerment of women and providers. We anticipate that our intervention will enhance providers’ empowerment by presenting them with monthly statistics promoting reflexivity on their practices, and this increased awareness of clinical practices will, in turn, enhance obstetricians’ and midwives’ sense of agency and self-determination in deciding on interventions during labor and delivery. From the women’s point of view, the study will detail the self-empowerment and professional support provided to women to choose the mode of delivery that better suits their needs. We will analyze the effect of the intervention on women’s self-esteem, knowledge, and sense of empowerment when deciding on the mode of delivery. In our analysis of empowerment, particular attention will be paid to the gender dimension, including the gender dynamics between different categories of providers and between providers and women and how gender norms shape values and decisions related to childbirth.

### Subgroup analyses

The integration of quantitative process measures into outcome datasets will contribute to understanding how implementation variability affects outcomes (on-treatment analyses) and to testing hypotheses arising from qualitative analyses. For example, time-series models with multigroup comparisons will enable us to conduct formal statistical tests comparing the level and slope of the primary outcome between different categories of healthcare facilities reflecting different levels of implementation, thereby quantifying the variation of effect size between subgroups [41] and revealing the mechanisms of impact. Additionally, outcomes between women in different socioeconomic categories (in terms of education, place of residence, place of birth, and wealth index) and between periods will be compared to assess the equity of the QUALI-DEC intervention.

### Knowledge transfer

In consultation with key stakeholders in each participating country, we will develop an innovative evidence-based knowledge transfer strategy [42, 43], adapted to each context. The key ingredients of this strategy will be training, implementation, and evaluation of a knowledge broker in each country who will facilitate the adaptation, dissemination, and exploitation of QUALI-DEC findings by key stakeholders (see Additional files 1, 2 and 3). As the implementation of knowledge brokering is very innovative, it will be the subject of an in-depth evaluation in order to generate knowledge about its processes and effectiveness. A specific research protocol for this part of the QUALI-DEC project will be published later.

## Discussion

There is an urgent need for interventions and implementation strategies that optimize the use of CS while improving health outcomes and satisfaction in LMICs. QUALI-DEC aims to test whether the audit and feedback, decision aid, and patient-centered care approaches supported by local OLs improve the quality of decision-making and perinatal outcomes.

The components of the QUALI-DEC intervention have been tested in randomized controlled trials (RCTs) aiming to reduce the overuse of CS [10]. However, the research reported to date has shown only modest effectiveness in reducing CS rates (Table 1). Evidence on the effect of implementing evidence-based clinical practice guidelines combined with A&F and opinion leaders was available from two RCTS, both from Canada [44, 45]. The effect size on CS rates was moderate to low. However, no research evidence was identified on the acceptability or the impact on equity of this type of intervention. Evidence on the effect of companionship was available from a meta-analysis of 26 RCTS [11]. The effect size on CS rates was moderate. A qualitative evidence synthesis identified various factors affecting successful implementation and sustainability of labor companionship [46]. Three RCTs conducted in the UK [47], Australia [48], and the USA [49] found that decision-support tools improved women’s knowledge and reducing decisional conflict about mode of delivery options, but had variable effects on their uptake of trial of labor or vaginal birth after cesarean section. However, there are no published randomized trials on the effect of decision aids on women without a previous cesarean section. A qualitative evidence synthesis suggests that women welcome new information and learning about childbirth which can mediate pregnant women’s concerns about risk. Women perceive educational interventions and decision-aid tools as a “starting point,” a springboard for seeking more information and for a more meaningful dialog with health professionals [50].

The reasons behind the limited success of nonclinical interventions to reduce unnecessary CS include the failure to acknowledge the multifactorial and complex nature of CS overuse and, accordingly, the failure to design multifaceted interventions. In addition, not enough emphasis has been given to the evaluation of the implementation strategies, which is a critical component underpinning effectiveness, particularly regarding complex and behavioral driven interventions. Our ambition is to implement a multifaceted intervention targeted at women, healthcare professionals, and organizations simultaneously. The study will generate evidence on the feasibility, acceptability, implementation, effectiveness, and equity of this intervention to reduce overuse of CS in various settings in LMICs and on approaches to overcome barriers to implementation. Our project will go beyond the state of the art for the following reasons. First, it will provide an exhaustive description of the barriers and facilitators to implementing the four components of the QUALI-DEC intervention in various settings under a rigorous formative research phase [19]. This information will help identify and systematically structure-specific determinants associated with implementation success. This is particularly true for the implementation of companionship during labor and evidence-based clinical practice guidelines combined with A&F and opinion leaders. Second, it will help explain what influences implementation outcomes and provide information on the overuse of CS in settings where the performance in terms of CS decision-making is currently undocumented. Third, it will integrate qualitative and quantitative data to strengthen the internal validity of the results. Combining the merits of multiple theoretical approaches, the QUALI-DEC project will offer a more complete understanding by providing a theory of change that could be adapted to different settings [29]. Fourth, it will analyze the scalability and transferability of the intervention to other contexts, a pressing issue considering the global rise in CS in the past few decades. Importantly, QUALI-DEC focuses on LMICs, where addressing the challenge of overuse has become a priority.

In conclusion, the findings from this pragmatic evaluation will be highly applicable to practitioners, service managers, and policy-makers who are tasked with implementing a nonclinical intervention to reduce unnecessary CS in LMICs. In addition, the findings will determine the effectiveness and cost-effectiveness of an innovative implementation strategy tailored to the needs of the local setting. This strategy aims to implement four active components that are expected to improve quality decision-making for the mode of birth so that only the women who need to have a CS undergo the procedure. The strategy will involve women, healthcare professionals, and organizations and will focus on how to best and most effectively implement these components, considering the local needs and resources in each country. Overall, our project will improve the appropriate use of CS and address several sustainable development goal targets, including improving maternal and neonatal health and reducing inequalities within and between countries.

## Supplementary information

**Additional file 1.** StaRI checklist.

**Additional file 2.** Extended cost-effectiveness analysis od QUALI-DEC.

**Additional file 3.** Knowledge transfer strategy.

## Data Availability

The data produced and published during the QUALI-DEC project will be accessible in Zenodo (https://www.zenodo.org/), a general-purpose open-access repository developed under the European OpenAIRE program and operated by the European Organization for Nuclear Research (CERN). Zenodo will allow the deposition of datasets, reports, and any other digital artifacts related to research. For each repository, a persistent DOI will be created to easily cite the stored items. The metadata of each record will be indexed and searchable directly in Zenodo’s search engine immediately after publishing.

## Abbreviations

A&F: Audit and feedback
CS: Cesarean section
DAT: Decision analysis tool
ECEA: Extended cost-effectiveness analysis
ICER: Incremental cost-effectiveness ratio
IDI: In-depth individual interview
IEC: Information, education, communication
ITSA: Interrupted time series analysis
LMIC: Low- and middle-income countries
MRC: Medical research council
OL: Opinion leader
OOP: Out-of-pocket
QUALI-DEC: QUALIty DECision-making by women and providers
REDCap: Research electronic data capture
TGCS: Ten group classification system
WHO: World Health Organization
