# Supplementary material for: Implementation and evaluation of nonclinical interventions for appropriate use of cesarean section in low- and middle-income countries: protocol for a multisite hybrid effectiveness-implementation type III trial
Source: Implement Sci. 2020 Sep 4;15:72. doi: 10.1186/s13012-020-01029-4 (PMC7650262; doi:10.1186/s13012-020-01029-4)
Supplement: Supplementary file 2 — Additional file 2. Extended cost-effectiveness analysis od QUALI-DEC. [file 13012_2020_1029_MOESM2_ESM.docx]

**Extended cost-effectiveness analysis of QUALI-DEC**

The extended cost-effectiveness analysis (ECAE) follows the same principles as the traditional cost-effectiveness analysis; however, ECEA allows policy-makers to account for both health and nonhealth outcomes when making decisions and thus to more effectively direct scarce healthcare resources towards specific policy objectives. Furthermore, ECEA assesses the health and financial consequences of policies, including financial risk protection and other disaggregated outcomes per a specific population stratum of interest.

Information on intervention costs will be collected at the national and facility levels. Information on individual costs will be collected from women in relation to the postpartum survey. Intervention costs will be assessed at two levels: (i) the national level (development of guidelines and training materials, quarterly visits, and coordination costs) and (ii) the healthcare facility level (time spent by health professionals and OLs on training activities, A&F, consumables, equipment, overheads, and capital costs) and collected during the intervention implementation. OOP costs will also be collected from women in relation to activities performed in evaluating effectiveness outcomes (postpartum survey) and will include the following: the formal and informal direct payment for medical care, direct nonmedical costs (i.e., transportation costs to seek care), and indirect costs (i.e., time and productivity losses, which can be translated into wages and foregone income). Costs for the eventual scale-up of the intervention will also be calculated. Health gains will be determined by evaluating changes in CS practices and perinatal outcomes and fed into the ECEA.

The ECEA will provide two main outputs: the *incremental* *cost-effectiveness ratio (ICER)* – the net costs per reduction in CS among low-risk women – will be computed; and *OOP averted* – this output can be referred to as ‘expenditures crowded out’ in the sense that the implementation of the QUALI-DEC intervention may lead to the ‘crowding out’ of these individual OOP costs. The distributional consequences (i.e., outputs) will be analyzed across distinct strata of the populations (e.g., socioeconomic status and geographical setting).
