# Supplementary material for: Implementation and evaluation of nonclinical interventions for appropriate use of cesarean section in low- and middle-income countries: protocol for a multisite hybrid effectiveness-implementation type III trial
Source: Implement Sci. 2020 Sep 4;15:72. doi: 10.1186/s13012-020-01029-4 (PMC7650262; doi:10.1186/s13012-020-01029-4)
Supplement: Supplementary file 3 — Additional file 3. Knowledge transfer strategy. [file 13012_2020_1029_MOESM3_ESM.docx]

**Knowledge transfer strategy**

In consultation with the Ministries of Health (MoH) of participating countries, WHO, and other stakeholders including professional obstetrician and midwifery organisations, we will develop an innovative evidence-based Knowledge Transfer (KT) strategy, adapted to contextual factors for each country. The key components of this strategy will be: (1) training and implementation of a knowledge broker in each country who will facilitate the adaptation, dissemination and exploitation of QUALI-DEC findings by key stakeholders; (2) capacity building of key stakeholders in KT strategies; and (3) national and international deliberative dialogues.

*Knowledge Brokering strategy.* Among the different options of KT strategies, knowledge brokering (KB) is one of the most promising because it involves an active face-to-face engagement of stakeholders by a trained person tailoring communication of evidence to each stakeholder [Dagenais 2016]. Thus, QUALI-DEC will implement KB strategies in each country. In each country, a knowledge broker will be recruited, trained and couched by KT researchers. The translation of three MOOCs into English will enable distance learning for all QUALI-DEC researchers and decision-makers as well as KB. The latter will also have a 5-day face-to-face training session. Trained knowledge brokers will identify, engage and connect with stakeholders and decision-makers; support sustainability; and create tailored knowledge products for each country.

*Capacity building in knowledge transfer.* To ensure the effectiveness of the KT strategy and the use of the project findings, QUALI-DEC will start its KT actions by strengthening the capacities of all stakeholders (researchers, health professionals, MoH managers, local and national decision-makers and journalists). Distance (MOOC) and face-to-face training workshops on KT will be offered. The objectives of the workshops will be for participants to become familiar with the basic concepts of KT; to learn about KT strategies, activities and tools (policy briefs, deliberative dialogues, infographics); to identify the factors that promote and hinder the use of evidence; and to develop the skills needed to prepare KT tools. Specifically, researchers will be trained on KT strategies and then supported to propare KT plans, hospital managers and decision-makers in the critical reading of scientific articles and the use of evidence for decision-making, and local journalists in the scientific process and production of articles to disseminate research results to the general audience.

*Deliberative dialogues.* Beyond the dissemination workshops that are useful for sharing research results, we will also create real policy dialogues to strengthen research use. Thus, the KBs will organize in each country a national workshop in the form of a deliberative dialogue [Ridde 2017] with all the key stakeholders (researchers, health professionals, MoH managers, women associations, professional associations, local and national decision-makers) to discuss research results and take decisions for implementation. Subsequently, an international deliberative dialogue workshop will be organized with WHO in Geneva, with representatives from each country and international organisations concerned by the results of QUALI-DEC, to maximize the impact of learnings from our study findings for other contexts and other global health organisations.

*Evaluation of KT strategy.*  We will conduct an evaluation of KT strategy on the use of evidence to influence decision making. The evaluation methodology will consist of multiple case studies with several levels of analysis, as recommended for complex interventions [Moore 2015]. The cases will be the KBs and all their activities. The evaluation will be conducted using a mixed-method approach: qualitative interviews conducted with respect to the dimensions of relevant KT frameworks (e.g. context, process, power relation, innovation, leadership, etc) [Durlak 2008; Damschroder 2009; Boyko 2012]; and quantitative questionnaires with participants in the deliberative dialogue workshops to measure their intention to use the research results through integration of planned behaviour theory and interpersonal behaviour theory.

**References**

1. Ridde V, Dagenais C. What we have learnt (so far) about deliberative dialogue for evidence-based policymaking in West Africa. BMJ Glob Health. 2017;2(4):e000432-e000432.
2. Moore GF, Audrey S, Barker M, et al. Process evaluation of complex interventions: Medical Research Council guidance. BMJ. 2015;350(mar19 6):h1258-h1258.
3. Durlak J, DuPre E. Implementation matters: a review of research on the influence of implementation on program outcomes and the factors affecting implementation. AM J Community Psychol. 2008;41(3-4):327-350.
4. Damschroder L, Aron D, Keith R, Kirsch S, Alexander J, Lowery J. Fostering implementation of health services research findings into practice: a consolidated framework for advancing implementation science. Implement Sci. 2009;4:50.
5. Boyko J, Lavis J, Abelson J, Dobbins M, Carter N. Deliberative dialoguesa as a mechanism for knowledge translation and exchange in health systems decision-making. Soc Sci Med. 2012;75:1938-1945.
